# Diversity of picoeukaryotes at an oligotrophic site off the Northeastern Red Sea Coast

**DOI:** 10.1186/2046-9063-9-16

**Published:** 2013-08-20

**Authors:** Francisco Acosta, David Kamanda Ngugi, Ulrich Stingl

**Affiliations:** 1 Red Sea Research Center, King Abdullah University of Science and Technology, 4700 KAUST, Thuwal 23955-6900, Saudi Arabia

**Keywords:** Picoeukaryotes, Red sea, Protists, SSU rRNA, Microbial diversity

## Abstract

**Background:**

Picoeukaryotes are protists ≤ 3 μm composed of a wide diversity of taxonomic groups. They are an important constituent of the ocean’s microbiota and perform essential ecological roles in marine nutrient and carbon cycles. Despite their importance, the true extent of their diversity has only recently been uncovered by molecular surveys that resulted in the discovery of a substantial number of previously unknown groups. No study on picoeukaryote diversity has been conducted so far in the main Red Sea basin-a unique marine environment characterized by oligotrophic conditions, high levels of irradiance, high salinity and increased water temperature.

**Results:**

We sampled surface waters off the coast of the northeastern Red Sea and analyzed the picoeukaryotic diversity using Sanger-based clone libraries of the 18S rRNA gene in order to produce high quality, nearly full-length sequences. The community captured by our approach was dominated by three main phyla, the alveolates, stramenopiles and chlorophytes; members of Radiolaria, Cercozoa and Haptophyta were also found, albeit in low abundances. Photosynthetic organisms were especially diverse and abundant in the sample, confirming the importance of picophytoplankton for primary production in the basin as well as indicating the existence of numerous ecological micro-niches for this trophic level in the upper euphotic zone. Heterotrophic organisms were mostly composed of the presumably parasitic Marine Alveolates (MALV) and the presumably bacterivorous Marine Stramenopiles (MAST) groups. A small number of sequences that did not cluster closely with known clades were also found, especially in the MALV-II group, some of which could potentially belong to novel clades.

**Conclusions:**

This study provides the first snapshot of the picoeukaryotic diversity present in surface waters of the Red Sea, hence setting the stage for large-scale surveying and characterization of the eukaryotic diversity in the entire basin. Our results indicate that the picoeukaryotic community in the northern Red Sea, despite its unique physiochemical conditions (i.e. increased temperatures, increased salinity, and high UV irradiance) does not differ vastly from its counterparts in other oligotrophic marine habitats.

## Background

Unicellular eukaryotic organisms are a ubiquitous and important component of marine ecosystems, which interacts closely with *Bacteria* and *Archaea* in trophic and biogeochemical cycles that shape the ocean’s biosphere. Yet, our current knowledge of their ecological roles and diversity in these ecosystems is surprisingly limited, especially compared to the other domains of life. After more than a decade of continuous surveys, we have only started to uncover the true extend of marine protistan diversity. The recent discoveries of novel groups, even up to the phylum level [1,2], whose ecological and physiological characteristics remain completely in the dark, only serves to reinforce the notion that small eukaryotes are one of the lesser known and studied components of marine ecosystems.

Among eukaryotes, organisms in the picoplankonic fraction are collectively called picoeukaryotes (PE). They are the smallest organisms in this domain, and are comprised of protists in the size range of 3 μm and smaller. PE are an important and often overlooked component of marine ecosystems and include phototrophic, heterotrophic and mixotrophic organisms. They constitute one of the main components of the photosynthetic picoplankton, which, under certain environmental conditions, can represent the main primary producers in surface waters [3-5]. Despite the fact that photosynthetic PE have a global average concentration of 10^2^-10^5^ cell ml^–1^ in the upper photic zone [6], which is one to two orders of magnitude lower than photosynthetic bacteria such as *Prochlorococcus* and *Synechococcus*, their contribution to carbon fixation, and thus to global ocean carbon budgets, is significant due to their high cell-specific carbon uptake rates and high carbon content [4,7]. In the Atlantic Ocean, picoeukaryotic phytoplankton are estimated to comprise approximately one fifth of the total biomass of this ocean, with a total biomass of 20-45 million tonnes carbon [8].

Heterotrophic (mostly bacterivorous) PE belonging to abundant groups such as alveolates and stramenopiles are also a vital link for the recycling of nutrients from the prokaryotic fraction to higher trophic levels in the marine microbial loop [9]. Likewise, interactions between PE and prokaryotes have further ecological implications, as bacterial abundances and community composition are strongly influenced by the predation pressure of PE [10]. Besides primary production and bacterivory, PE can also influence different trophic levels through parasitic and mutualistic symbiotic associations [11].

The Red Sea is a unique marine environment with distinctive physico-chemical conditions including year-round high levels of irradiance, high temperature and salinity levels, and low nutrient concentrations-particularly in the northern area of the basin. However, up to date most of the basin remains underexplored in terms of its microbial diversity. The exception is the Gulf of Aqaba, a semi-enclosed basin in the northern part of the Red Sea, which, while still being considered an oligotrophic environment, has a more pronounced seasonality and higher primary production and chlorophyll levels than the main Red Sea basin due to strong convective mixing processes [12].

Recent in-depth studies have made significant progress towards an understanding of the diversity and distribution of prokaryotic communities in the main basin of the Red Sea [13,14], which seem surprisingly similar to those of other, less extreme marine oligotrophic sites. In contrast, much less is known about the eukaryotic microbiota and its ecological roles, with most published studies having focused mainly on photosynthetic PE. Only few studies have been conducted to investigate and characterize eukaryotes both in the Gulf of Aqaba [12,15] and in the main Red Sea basin [16-18]. Among these, only a few have focussed on the taxonomic diversity of these groups [15,19].

Microbiological research in the Red Sea is especially relevant now, since future global climate changes are predicted to include higher surface temperatures accompanied with increased stratification and nutrient deficiency of waters in the euphotic zone of the world’s oceans [20]. Such conditions are already naturally present in the Red Sea basin, and similar trends have been reported from the Arctic Ocean, where they favour the prevalence of small phytoplankton, to the detriment of larger primary producers [21]. Thus, studies on the diversity of PE communities in the Red Sea may provide us with insights on how microbes in the world’s oceans will change in the following decades.

This study was therefore designed to investigate the PE diversity in surface waters of the northeastern Red Sea. The selected sampling sites are part of a region characterized by relatively high temperatures (18°C-35°C) [22], high salinity (~40 psu) [22] and year-round stratification, coupled with low nutrient concentrations [23]. The underlying environmental conditions of the chosen area can be considered extreme for marine ecosystems and include an area that has not been covered in other picoeukaryotic surveys. We used ribosomal SSU-based clone libraries, which have been employed extensively in a multitude of environments, from polar to tropical seas as well as a large number of extreme habitats, in order to provide high quality, nearly full-length sequences.

## Results

In order to capture most of the eukaryotic diversity present in the picoplankton, we created two clone libraries for both the 5.0-1.2 μm fraction (large-sized eukaryotic fraction) and the 1.2-0.1 μm fraction (small-sized eukaryotic fraction), using general eukaryotic primers. Clone libraries of the large-sized and small-sized eukaryotic fractions produced 253 and 56 high quality nearly full-length sequences respectively, after removal of all low-quality, unassembled and potentially chimeric sequences. These clustered into a total of 118 operational taxonomic units (OTUs) at a sequence distance threshold of 0.02. Individually, the large-sized fraction had 101 OTUs, whereas 21 OTUs were observed in the small-sized eukaryotic fraction; there were four common OTUs present in both libraries.

While samples for each fraction were taken from two different sites, they constitute neighbouring locations with very similar physico-chemical properties and bacterial community structures Table 1, [24]. Despite these similarities, we have refrained from drawing any conclusions based on the presence or absence of taxa in each sampling site, focusing instead in the general picoeukaryotic diversity of the sampled region.

**Table 1:** Sampling sites and their physical and environmental characteristics

**Site**	**Coordinates**	**Temperature (°C)**	**Salinity (psu)**	**Chl a (mg m**^**-3**^**)**	**Oxygen (mg l**^**-1**^**)**	PIC* (mmol m^**–3**^**)**	**POC**^**#**^**(mg m**^**-3**^**)**	**Fraction surveyed**
1	25.17 N 36.89 E	25.32	39.48	0.15	4.25	0.12	49.7	5.0-1.2 μm
2	25.89 N 36.49 E	25.05	39.72	0.16	4.26	0.14	50.73	1.2-0.1 μm

*PIC particulate inorganic carbon, ^#^*POC* particulate organic carbon.

Initial analysis regarding the community composition for both libraries showed that the small-sized eukaryotic fraction was dominated by metazoan sequences (~60% of all clones). The presence of these sequences is considered a normal contaminant in almost all unicellular eukaryotic surveys, including those of marine picoplankton [25,26], and it is the result of genetic material from cell debris of zooplanktonic organisms. Additionally, there were nearly no unique sequences in the smallest fraction, as most of the PE sequences belonged or were highly similar to OTUs found in the large size fraction.

**Figure 1:**
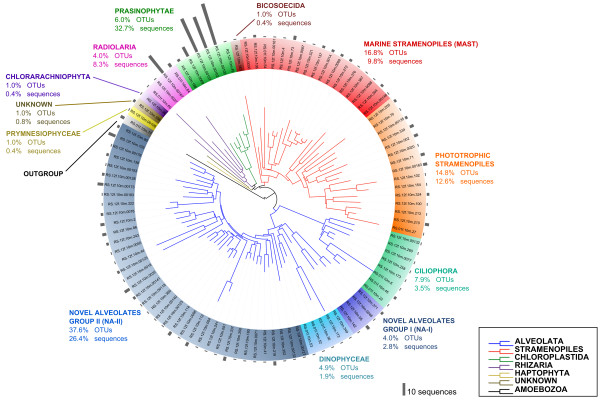
**Community composition of picoeukaryotes based on a maximum likelihood phylogenetic tree of all representative picoeukaryotic OTU (98% similarity) sequences.** Colored branches in the tree represent the main eukaryotic phyla, while the colored sectors indicate the different taxa to which the sequences could be confidently assigned. Grey bars at the outer ring indicate the relative abundance of sequences within each OTU. An amoebozoan OTU obtained in this study was used as outgroup [GenBank: KC583130].

In order to analyse the total diversity of PE, we pooled the sequences from both fractions, retrieving in total 101 prospective PE OTUs; their taxonomic affiliation, as well as their diversity and sequence abundances are summarized in Figure 1. The community is dominated by sequences related to alveolates, stramenopiles and prasinophytes, which together account for 90.1% of all sequences and 93.0% of all PE OTUs (Table 2, Figure 1). Rarefaction curves for the large-sized eukaryotic fraction library using different OTU clustering criteria are shown in Additional file 1: Figure S1. Diversity analyses on the same library showed that a number of protistan OTUs probably remain undetected, since total OTU richness is estimated to range between 142-261 and 150-275 using the nonparametric Chao1 and ACE estimators, respectively. These values are in good agreement with the estimated Good’s coverage (76.6% of diversity was reached), although these estimations are undoubtedly confounded by the presence of non-PE sequences. The analysis of the same dataset after the removal of metazoan sequences showed slightly lower diversity values (130-246 for Chao1, 131-234 for ACE). Based on these values, we believe that we have captured most of the main PE groups in the sampled sites and provide for the first time high quality, nearly full-length 18S rRNA gene sequences of PE from the Red Sea. Nevertheless, the use of other approaches, such as amplicon sequencing using next generation sequencing, would help to retrieve the complete diversity of PE in the sampled region. Still, the data presented here will be invaluable for the alignment of short sequence reads and assignment of OTUs.

**Table 2:** Sequence abundance and diversity of picoeukaryotic taxa found in this study

**Phylogenetic groups**	**Number of sequences**	**Number of OTUs**
Alveolata		
Dinoflagellates	5 (1.9%)	5 (4.9%)
Ciliates	9 (3.5%)	8 (7.9%)
NA-I	7 (2.8%)	4 (4.0%)
NA-II	67 (26.4)	38 (37.6%)
Chlorophyta		
Mamiellales (*Ostreococcus tauri*)	30 (11.8%)	2 (2.0%)
Mamiellales (*Micromonas pusilla*)	28 (11.0%)	2 (2.0%)
Mamiellales (*Bathycoccus prasinos*)	21 (9.1%)	1 (1.0%)
Clade VII	2 (0.8%)	1 (1.0%)
Stramenopiles		
Chrysophytes	15 (5.9%)	8 (7.9%)
Dictyochophytes	7 (2.8%)	3 (3.0%)
Pinguiophytes	1 (0.4%)	1 (1.0%)
Bolidophytes	5 (1.9%)	2 (2.0%)
Unclassified	4 (1.6%)	1 (1.0%)
Bicosoecids	1 (0.4%)	1 (1.0%)
MAST	25 (9.8%)	17 (16.8%)
Rhizaria		
Radiolarians	21 (8.3%)	4 (4.0%)
Chloroarachniophtyes	1 (0.4%)	1 (1.0%)
Haptophyta		
Prymnesiophytes	1 (0.4%)	1 (1.0%)
Unknown	2 (0.8%)	1 (1.0%)
Non-PE sequences	55	17

### Alveolates

Alveolates were the most abundant and diverse group in our libraries, encompassing 34.6% of all PE sequences and 54.4% of all OTUs. Phylogenetic trees for this group are presented in Additional files 2 and 3: Figures S2 and S3. The overwhelming majority belonged to the Marine Alveolates (MALV) groups, which comprise almost exclusively environmental sequences belonging to so far uncultured organisms.

Most alveolate sequences (76.3%) clustered within the MALV-II group, which was also the most taxonomically diverse group in our study. It consisted of 38 representative OTUs distributed among 18 distinctive groups. The most abundant clades in our samples were Clades 10 + 11 (9 OTUs) and Clade 6 (3 OTUs). The rest of the OTUs were distributed in low abundance among different clades. Almost all of these clades are composed of sequences stemming from marine surface waters, with the Red Sea clones having high similarities to sequences from the Mediterranean Sea, the Sargasso Sea and the coastal Pacific Ocean. However, almost 18% of all MALV-II sequences (12 sequences, distributed in 6 OTUs) found in this study do not appear to be related to any previously described clades, neither do they associate with other environmental clones. Some of these sequences cluster together as phylogenetically distinct groups, which we have named RS1 and RS2 (Additional file 2: Figure S2). The seven clones from the MALV group I (MALV-I) clustered among Clades 1 and 5 (Additional file 3: Figure S3). The rest of alveolate sequences belonged to non-MALV organisms, namely dinoflagellates from the orders *Gymnodiniales* and *Gonyaulacales* and spirotrichid ciliates (Additional file 3: Figure S3).

### Stramenopiles

Stramenopiles were the second most diverse group in our clone library, containing 33 OTUs and 22.8% of the overall number of sequences. Almost half of these OTUs (45%) belonged to potentially photosynthetic taxa, namely organisms in the classes *Chrysophyceae*, *Bolidophyceae*, *Dictychophyceae*, *Pinguiophyceae* and a non-described phototrophic group (Table 2, Additional file 4: Figure S4).

The highest diversity for the stramenopiles was observed within *Chrysophyceae* (8 OTUs), with sequences belonging to the hitherto uncultured clades H, G and I (as defined by del Campo et al. [27]). Other groups were less abundant, and were highly similar to described species. Bolidophyte sequences were closely related to *Bolidomonas pacifica*, and pinguiophyte-like sequences clustered with those of the genus *Phaeomonas*. The *Dictyochophyceae* were distributed into two clusters; one containing sequences from *Florenciella parvula*, and the other forming a monophyletic group within the order *Pedinellales* with low similarity (<94%) to available sequences.

Four of our sequences, which were present in both size fractions, formed a single OTU (OTU-92) that clustered with a number of environmental sequences into a sister group of the *Pinguiophyceae*. This group includes the cultured strain RCC853, an unclassified photosynthetic stramenopile isolated from the southeast Pacific [28]. Unfortunately, as mentioned in the original paper, this strain has been lost, preventing further analysis regarding the taxonomic classification of these organisms.

The rest of the stramenopile sequences (17.8% of all PE OTUs) fell within presumably heterotrophic groups. Except for a single bicosoecid sequence, most belonged to the Novel Marine Stramenopiles (MAST) groups that were initially described by Massana et al [29]. Most of our MAST sequences (75%) fall in clades MAST-4,-7 and-3, in decreasing order of abundance. Other groups include MAST-6,-11,-9 and-12. Similarly to the NA-associated sequences, our clones also had high similarities to sequences from the Pacific Ocean and the Sargasso and Mediterranean Seas.

### Chlorophytes

Chlorophytes represented the second most abundant group in our study, comprising 32.7% of both of our libraries (Table 2). They were almost exclusively composed of prasinophytes (Table 2, Additional file 5: Figure S5). Despite their sequence abundance, they only contributed to 6% of the total picoeukaryotic OTUs, indicating a low genetic diversity of members of this group.

A third of the clones that fell within the order *Mamiellales* belong to the species *Micromonas pusilla*, making it the most abundant species in our clone libraries (11.8% of all sequences). These sequences show a high micro-diversity, and cluster within four different clades (A.BC.1, A.A.2, B._.4 and B.E.3.), based on the classification of Worden [30].

We found a similar number of *Ostreococcus*-related sequences belonging to Clade OII, an abundant clade associated with warm and relatively saline waters [31], consistent with conditions in our sampling sites. Clade OII is also presumed to be the only clade present in the Indian Ocean [32]. Most of the other *Mamiellales* sequences were closely affiliated to *Bathycoccus prasinos*.

### Other taxa

The other taxa represented in the clone libraries were found in low abundances (Additional file 5: Figure S5). Rhizarians were represented by five different OTUs, four of which were affiliated with radiolarians. Two of them were related to known radiolarian families, *Plagoniidae* and *Litheliidae*, while the other two clustered with environmental clades without cultured relatives (RAD-III and RAD-A). The fifth rhizarian OTU clustered closely to the chloroarachniophyte *Minorisa minuta*. Only one haptophyte sequence was found in our study, belonging to the genus *Chrysochromulina*, showing high similarity to *C. scutellum.*

Finally, one OTU (OTU-40) could not be assigned to any known eukaryotic group, even after extensive phylogenetic analysis. BLAST analysis shows them as related to a single environmental sequence found in surface waters of an unpolluted bay in the western Pacific [33] with a 95% sequence identity. Within this novel clade, there are other environmental sequences from environments such as the Norwegian Sea, the English Channel and the Southern Ocean, suggesting that this is a rare but widely distributed group.

## Discussion

Despite the relatively harsh conditions of the Red Sea, our study showed a typical oligotrophic community of small eukaryotes in the northeastern Red Sea, containing representatives from most major marine picoeukaryotic phyla. Alveolates, stramenopiles and prasinophytes were the most abundant taxa in our libraries, which is in agreement with report of other oligotrophic marine environments using similar methodologies [34]. Despite the low abundance of eukaryotic cells in the basin [17,23], richness values are consistent with those of previous studies done using comparably sized clone libraries [25,35]. The main eukaryotic groups in terms of diversity were alveolates and stramenopiles, which together account for 87% of all our PE OTUs. Most of the sequences of these taxa belong to (mostly) uncultured groups such as MALV-II and MAST, which contain few described organisms and whose physiological and ecological characteristics are still mostly unknown. Low abundance groups included sequences related to *Radiolaria*, *Cercozoa* and *Haptophyta*.

### Phototrophic taxa

Previous studies have shown that picoplanktonic organisms are the dominant component of the phytoplankton in the Gulf of Aqaba and the northern Red Sea, contributing up to 77% of the primary productivity [36]. Our results corroborate a high abundance of phototrophic PE, and also show a high phylogenetic diversity of the picoeukaryotic fraction in the surface waters of the northern Red Sea. The high diversity of photosynthetic PE probably reflects the presence of multiple micro-niches for primary production in the physically and chemically distinct euphotic zone of the Red Sea basin.

Previous research on plankton in the Red Sea has focused on the picophytoplankton, due to their prominent ecological role in primary production and the diagnostic ease of detecting and identifying cells using their characteristic pigments. These studies have shown that, while photosynthetic PE play an important ecological role influenced by seasonality in areas like the Gulf of Aqaba [15] and the Somalian coast [19], their contributions in terms of relative and total abundance in the Red Sea is much lower and often surpassed by prokaryotic organisms [17]. One likely explanation for this are the lower nutrient concentrations in the stratified northern Red Sea, which favor smaller and more nutrient-efficient species such as *Prochlorococcus* and *Synechococcus*, the former being particularly abundant in the photic zone of the Red Sea [14,23]. In the northern Red Sea, Sommer et al. [17] estimated an average of 2.66 × 10^3^ eukaryotic cells ml^–1^ in the phytoplankton during early spring, accounting for roughly 1% of the total microbial cell count. Similarly to reports from the Gulf of Aqaba, this proportion seems to increase during winter, with PE accounting for approximately 20% of the total phytoplankton cell abundance [23]. Pigment-based studies in which PE were classified in the southern part of the Red Sea, which is characterized by higher temperatures and lower salinity than in the North, showed the presence of prasinophytes, prymnesiophytes and dinoflagellates [19].

Considering the paucity of molecular-based studies to classify PE in the Red Sea, our study sheds more light onto their taxonomic diversity in the basin by uncovering a substantial number of taxa, including several hitherto undocumented groups. Phytoplanktonic taxa seem to be especially diverse and abundant at the sampled site. Roughly 46% of all our PE sequences were putatively phototrophic stramenopiles, prasinophytes and haptophytes, which contrasts with studies in other regions using the same methodologies, where sequences of potentially heterotrophic organisms dominate [25,32].

*Prasinophyceae*-related sequences were the most abundant group in our libraries, constituted almost completely by *Mamiellales* (*Micromonas* sp., *Ostreococcus* sp. and *Bathycoccus* sp.) Prasinophytes have been reported in the southern Red Sea [19], which is characterized by a more eutrophic mixed layer compared to the northern regions, owing to nutrient inputs from the Indian Ocean. Sequences associated with these three species were present in the large-sized fraction in similar numbers, presumably indicating the co-existence of populations with roughly similar abundances since they all possess similar SSU copy numbers [37]. These species are usually differentially distributed along nutrient-and depth-dependant gradients [32], with periodic alternations of dominance between them [30,38].

The other main group constituting the phytoplankton are the photosynthetic stramenopiles, the most abundant being the *Chrysophyceae* (7.8% of all OTUs). Numerous species from this group are heterotrophic, and the trophic status of most non-described clades within this group, such as the ones found in this study, is still unknown [27]. However, members of *Chrysophyceae* have been reported as important components of the phytoplankton in the Gulf of Aqaba, where they may account for up to 20% of the total number of cells [15]. Similarly, plastid surveys in surface waters of the nearby Arabian Sea were dominated by sequences of *Chrysophyceae*, and their presence was correlated with high irradiance [39]. This group is also widespread in other marine environments, for example the gyres in the Pacific [26] and Atlantic [40] oceans, where they play an important role in oceanic primary production [7]. While there is a notable diversity within the group, with at least ten different clades described [27], only a few of these seem to occur in nutrient-poor conditions. Phylogenetically, our *Chrysophyceae*-related sequences belong to the so far uncultured clades G, H and I, which are the same groups as reported in the Pacific and Atlantic Oceans.

When compared to the eukaryotic communities of other nutrient-poor regions, one key group that is absent in our sample are the prymnesiophytes. These photosynthetic haptophytes constitute one of the main groups of the picophytoplankton [7] and have been found in the Gulf of Aqaba [15] and the southern part of the Red Sea [19]. However, their absence might be due to a methodology bias, since it is known that PCR amplification has a bias against GC-rich sequences like the 18S rRNA gene of the prymnesiophytes, leading to their under-representation in clone library-based studies [32,41].

### Heterotrophic taxa

Novel alveolates are the most abundant heterotrophic group in our clone libraries, with more than a quarter of these sequences clustering with the MALV-II clade. This dominance of MALV-II presents a situation similar to marine environments such as the English Channel [25] and the Sargasso Sea [42], where MALV-II dominated picoeukaryotic communities. However, in the neighbouring more eutrophic Indian Ocean, MALV-I clade predominates [32].

This is the first study documenting the presence of novel alveolates in the Red Sea where they represent an important group both in terms of abundance and diversity. While the ecological role of most organisms within MALV-II is as yet undetermined, they are thought to be parasites of other microorganisms, including ciliates, dinoflagellates, and rhizarians [43]. They were not only one of the most abundant groups in our library, but also the most diverse, covering a wide range of clades. The diversity and relative abundance of MALV-II clades found in the Red Sea seems to be in accordance to those found in surface waters of multiple marine regions, with the exception of the absence of Clade 1, which is an abundant group in other marine environments such as the Mediterranean Sea and the Atlantic Ocean [43].

Among MALV-II, some OTUs were related to *Amoebophrya* sp.*,* the only described species of this group. *Amoebophrya* is presumably composed of intracellular parasites of other protists, mainly dinoflagellates. Species closely related to known hosts of *Amoebophrya* have been observed in abundance in the Red Sea [16] and therefore it is highly likely that the sequences that we retrieved belong to organisms that parasitize these populations. While we cannot make inferences about the ecological significance of these groups, since most MALV sequences from environmental surveys are thought to come from spores [44] and do not represent active organisms, our results still hint at a high diversity of parasitic relationships between protists in the sampled region. While showing similarities to other marine regions, the Red Sea does contain a characteristic assemblage of MALV-II clades, including the presence of a number of OTUs that could not be assigned to any described clade. Given the peculiar physiochemical conditions of the Red Sea (e.g. high temperature, increased osmotic stress due to high salinity) and its relative isolation, some of these could belong to novel groups or ecotypes within MALV-II. This is particularly plausible for groups like RS1, which contained multiple sequences clustering into various OTUs and showed high bootstrap support in our phylogenetic analysis.

The other main heterotrophic taxa are stramenopiles. Small heterotrophic flagellates have been observed in the northern Red Sea [17], occurring at densities of up to 160 ± 70 cells ml^-1^[16]. Here, we describe the taxonomic affiliation of some of these organisms for the first time. The major flagellate taxa were the MAST groups, which account for 9.8% of our clone libraries. Most of the MAST clades in our libraries are typical representatives of open ocean environments, and the more abundant groups that we detected (MAST-4,-3 and-7) account (along with MAST-1) for up to 74% of novel stramenopile sequences in marine environments [29]. These organisms perform a variety of trophic roles, including grazing and herbivory, although bacterivory seems to be predominant [45,46]. Bacterivores in the Red Sea have a huge number of prospective prey species, including common oligotrophic bacteria such as the SAR11 clade and cyanobacteria. Single cell sequencing has shown a tentative association between MAST-4 and *Candidatus* Pelagibacter ubique [47], a member of the SAR11 clade, the single most common bacterial group in the Red Sea [14]. As MAST-4 is the most common taxon in our library associated with bacterivory, a potential ecological relationship between these groups of marine microorganisms in the Red Sea is highly likely.

Given the diversity of MAST clades present in the site, they probably prey on a wide diversity of bacterial species. Potential preys may not be limited to prokaryotes only but also other eukaryotes, since MAST have also been shown to be capable of ingesting *Micromonas pusilla* and *Ostreococcus* sp. cells [46], which are also abundant in the sampled sites.

Only one member of the MAST has been reported previously in the Red Sea: *Solenicola setigera*[48]. This species has cells of 4-7 μm length and is the only described member of the MAST-3 group [49]. It is a common organism in the Gulf of Aqaba, where it grows as an epiphyte of the diatom *Dactyliosolen mediterraneus* and can reach densities of several thousand cells per liter of seawater during the spring bloom [48]. Despite the fact that MAST-3 sequences were found in our libraries, no sequences related to *S. setigera* were found, probably due to the considerable environmental differences between the Gulf of Aqaba and the main basin of the northern Red Sea.

## Conclusion

As with other marine environments, the application of PCR clone libraries has uncovered a diverse picoeukaryotic community in the Red Sea. Even though our diversity coverage of this environment was limited, we could identify a wide range of groups, including some putatively novel taxa. The sampled region is characterized by a substantial diversity of primary producers in agreement with communities found in most other highly oligotrophic open-ocean locations. The heterotrophic fraction is composed mainly by parasitic novel alveolates and bacterivores from the MAST groups, both taxa being reported from the northern Red Sea for the first time. This study provides an important first step in the phylogenetic characterization of the eukaryotic component of the Red Sea microbiota. Subsequent studies will undoubtedly lead to a better understanding of their ecological roles in this unique environment.

## Methods

### Sampling

DNA material used in this study was available from a previous expedition [14]. Briefly, 20 L of seawater were taken by Niskin bottles from 10-m depths at two coastal sites off the northeastern coast of the Red Sea in March 2010: Site I (25.17°N 36.89°E) and Site II (25.89°N 36.49°E). Samples were pre-filtered using a peristaltic pump through a 5 μm pore size mixed-cellulose ester membrane filter and then consecutively through two filters with pore sizes of 1.2 and 0.1 μm. The use of a 0.1-μm filter instead of bigger sized filters (0.2-0.6 μm ) allowed for the capture of smaller bacteria on the filters for a different study [14], but might have increased the percentage of metazoan debris compared to other studies on PE. DNA was extracted from each of these using the protocols described in the respective publication [14].

### PCR and clone library construction

To construct clone libraries, 18S rRNA genes were PCR-amplified from DNA extracts from Site I (1.2 μm filter) and Site II (0.1 μm filter) using the universal eukaryotic primers EukA (5′-AACCTGGTTGATCCTGCCAGT-3′) and EukB (5′-TGATCCTTCTGCAGGTTCACCTAC-3′) [50]. The used filters capture all PE, as well as the smallest eukaryotes of the nanoplankton. The PCR reaction contained 2 μl of template DNA, 4 μM of each primer, 8 mM dNTPs, 5 μl of 10× ThermoPol Reaction Buffer (BioLabs) and 0.25 μl of TaqDNA Polymerase (BioLabs). The PCR program consisted of an initial denaturation step at 95°C for 5 min, 30 cycles of 30 s at 94°C, 90 s at 56.5°C, and 90 s at 72°C, and a final elongation step of 30 min at 72°C. All PCR reactions were done in triplicate using an Eppendorf Mastercycler Pro. PCR products were quality checked using 1% agarose gel electrophoresis and purified using the Qiagen MinElute PCR purification Kit. Purified PCR products from all three reactions were then separately cloned into the pCR®2.1-TOPO vector (Invitrogen) as per the manufacturer’s TOPO TA Cloning Kit protocol. Colonies with the correct insert size were then bi-directionally sequenced with M13 primers on an ABI 3730 × l DNA Analyzer in the Genomics Core Lab Facility at KAUST (Thuwal, Saudi Arabia).

### Community analysis

The raw sequences were pre-processed and trimmed using Sequencher 5.0 (Gene Codes Corporation) using default parameters. All low-quality or unassembled sequences were removed from further analysis at this point. Potential chimeric sequences were removed using a combination of three methods: (1) Bellephoron [51], (2) ChimeraSlayer [52], and (3) Uchime [53] packages as implemented in Mothur 1.21.1 [54]. BLAST [55] was also used to ascertain the taxonomy of sequences deemed chimeric based on the phylogenetic affiliation of the best hits for the forward and reverse sequences of any individual contig. Using this methodology, we detected 24 chimeric sequences from a total of 330 sequences (7.3%). Pairwise alignment of the high quality sequences were done based on the SILVA reference alignment (http://www.mothur.org/wiki/Silva_reference_files). Determination of Operational Taxonomic Unit (OTU) with a clustering criterion of 98% similarity, community composition, and taxonomic assignments were done in Mothur. Diversity values were determined using the inverted Simpson diversity index, and coverage was determined using Good’s coverage estimator [56]. Estimations for non-parametric species richness were done using the Chao1 and ACE estimators as implemented in Mothur. Sequences have been deposited at GenBank with the Accession Numbers GenBank: KC582877 to GenBank: KC583185.

### Phylogenetic analysis

All 309 non-chimeric sequences were aligned using the online automatic SILVA Incremental Aligner SINA v1.2.7 (http://www.arb-silva.de/aligner), and imported into the ARB phylogenetic software package v5.2 [57]. Phylogenetic trees were built using nearly full-length 18S rRNA gene sequences with 1076-1808 bp in length. Shorter sequences were added later to the complete trees using the parsimony option as implemented in ARB. Trees were built using two methods: the Maximum Likelihood method (based on the GTR model with PHYML [58]), and the Neighbour Joining method [59] (based on Jukes-Cantor distance correction matrix). Bootstrap values were calculated for both methods from 1000 replicates.

## Competing interests

The authors declare that they have no competing interests.

## Authors’ contributions

FA participated in the sequence retrieval, carried out the sequence alignment and phylogenetic analysis, participated in the community analysis and drafted the manuscript. DKN participated in the sequence retrieval and community analysis and helped to draft the manuscript. US initiated the study, participated in its design and coordination, and helped to draft the manuscript. All authors read and approved the final manuscript.

## Supplementary Material

Additional file 1: Figure S1 Rarefaction analyses of the clone library of the larger size fraction for different OTU clustering criteria. Click here for file

Additional file 2: Figure S2 Phylogenetic tree of representative MALV-II OTUs. Sequences from this study are shown in red color, with the number in brackets denoting the sequences counts per OTU. Bootstrap values for both maximum likelihood and neighbour-joining methods are indicated at the branch nodes as open (>50%) and closed circles (>90%). An asterisk denotes partial 18S rDNA sequences. Sequences from Acanthamoeba castellanii [GenBank:U07413] and Hartmannella vermiformis [GenBank:AF426157] were used as outgroup (not shown). Click here for file

Additional file 3: Figure S3 Phylogenetic tree of representative alveolate OTUs, excluding MALV-II OTUs. Sequences from this study are shown in red color, with the number in brackets denoting the sequence counts per OTU. Bootstrap values for both maximum likelihood and neighbour-joining methods are indicated at the branch nodes as open (>50%) and closed circles (>90%). An asterisk denotes partial 18S rDNA sequences. Sequences from Acanthamoeba castellanii [GenBank:U07413] and Hartmannella vermiformis [GenBank:AF426157] were used as outgroup (not shown). Click here for file

Additional file 4: Figure S4 Phylogenetic tree of representative stramenopile OTUs. Sequences from this study are shown in red color, with the number in brackets denoting the sequences counts per OTU. Bootstrap values for both ML and NJ methods are indicated at the branch nodes as open (>50%) and closed circles (>90%). An asterisk denotes partial 18S rDNA sequences. Sequences from Acanthamoeba castellanii [GenBank:U07413] and Hartmannella vermiformis [GenBank:AF426157] were used as outgroup (not shown)*.* Click here for file

Additional file 5: Figure S5 Phylogenetic tree for representative eukaryotic OTUs not related to alveolates or stramenopiles. Sequences from this study are shown in red color, with the number in brackets denoting the sequences counts per OTU. Bootstrap values for both maximum likelihood and neighbour-joining methods are indicated at the branch nodes as open (>50%) and closed circles (>90%). An asterisk denotes partial 18S rDNA sequences. Sequences from Acanthamoeba castellanii [GenBank:U07413] and Hartmannella vermiformis [GenBank:AF426157] were used as an outgroup (not shown). Click here for file
