# Supplementary figures and images for: Diversity of picoeukaryotes at an oligotrophic site off the Northeastern Red Sea Coast
Source: Aquat Biosyst. 2013 Aug 20;9:16. doi: 10.1186/2046-9063-9-16 (PMC3766091; doi:10.1186/2046-9063-9-16)

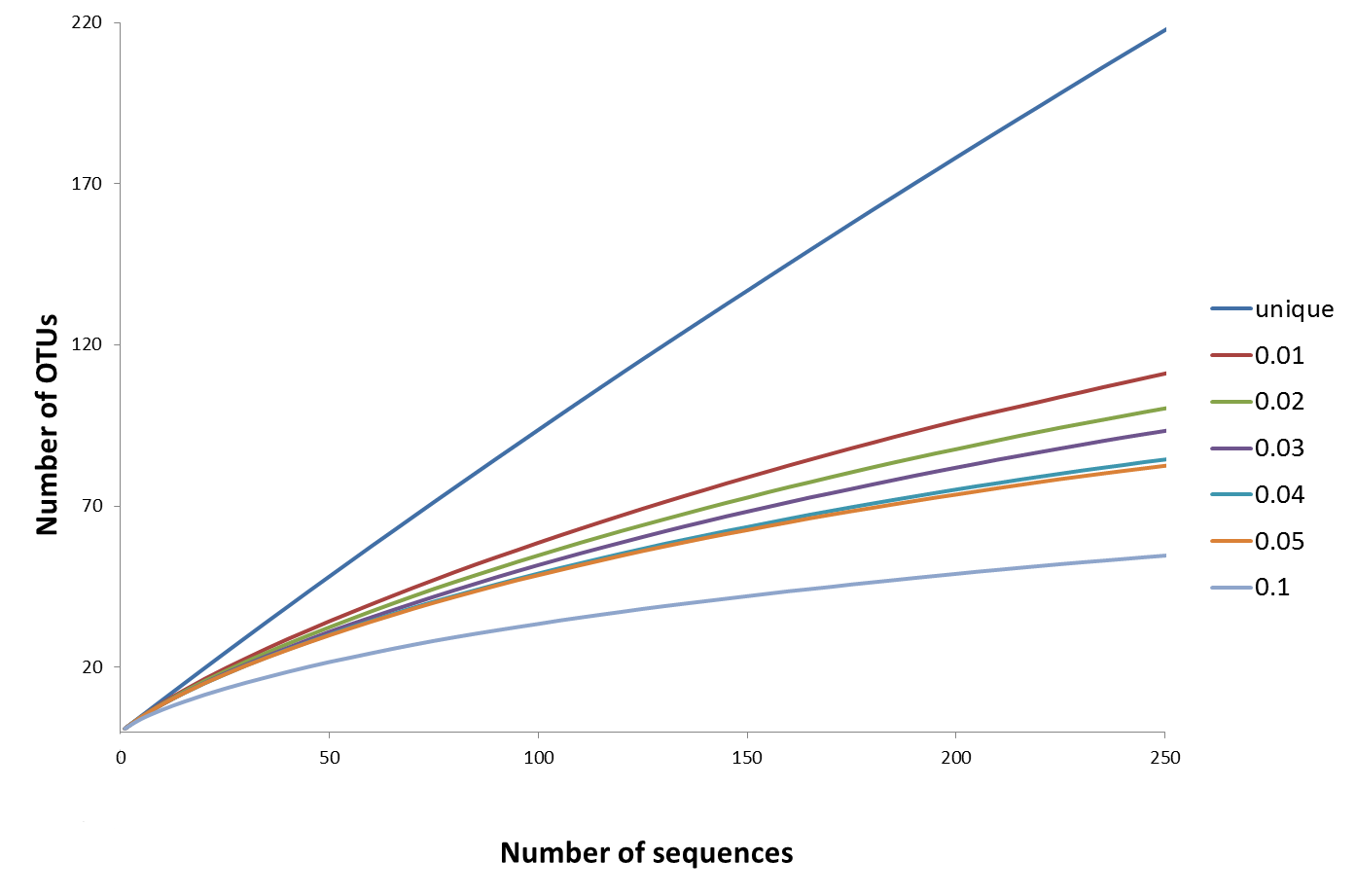

Supplement: Additional file 1: Figure S1 — Rarefaction analyses of the clone library of the larger size fraction for different OTU clustering criteria. [file 2046-9063-9-16-S1.png]

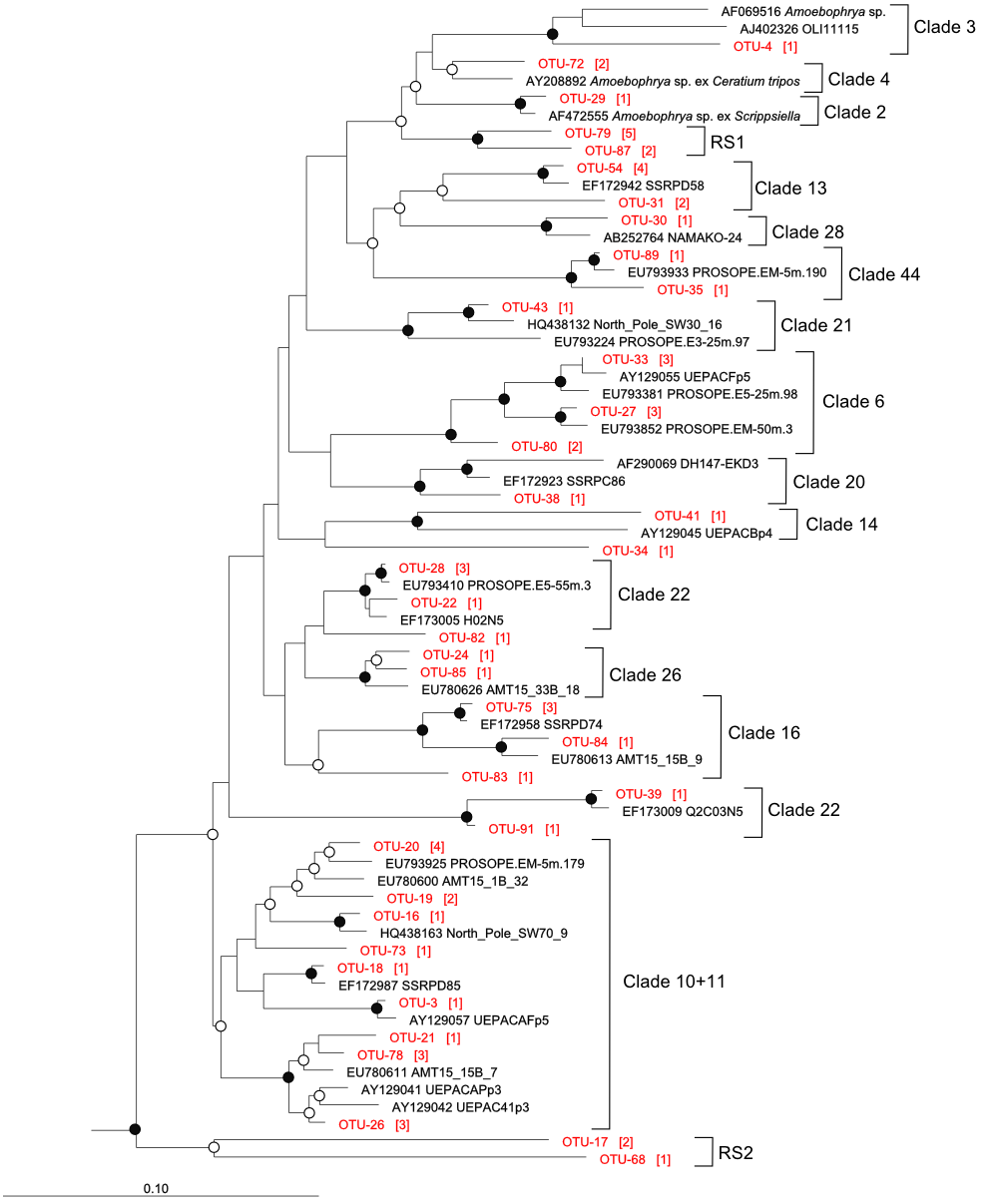

Supplement: Additional file 2: Figure S2 — Phylogenetic tree of representative MALV-II OTUs. Sequences from this study are shown in red color, with the number in brackets denoting the sequences counts per OTU. Bootstrap values for both maximum likelihood and neighbour-joining methods are indicated at the branch nodes as open (>50%) and closed circles (>90%). An asterisk denotes partial 18S rDNA sequences. Sequences from Acanthamoeba castellanii [GenBank:U07413] and Hartmannella vermiformis [GenBank:AF426157] were used as outgroup (not shown). [file 2046-9063-9-16-S2.png]

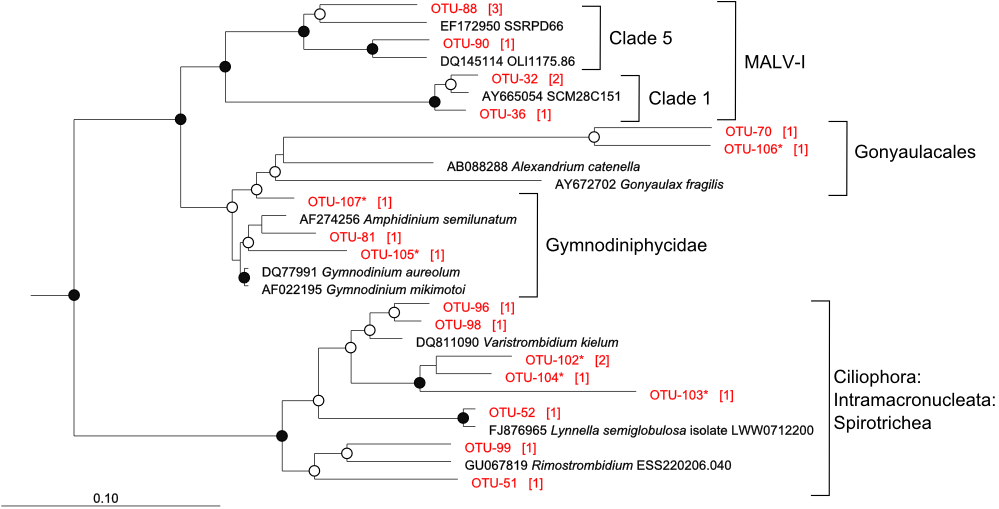

Supplement: Additional file 3: Figure S3 — Phylogenetic tree of representative alveolate OTUs, excluding MALV-II OTUs. Sequences from this study are shown in red color, with the number in brackets denoting the sequence counts per OTU. Bootstrap values for both maximum likelihood and neighbour-joining methods are indicated at the branch nodes as open (>50%) and closed circles (>90%). An asterisk denotes partial 18S rDNA sequences. Sequences from Acanthamoeba castellanii [GenBank:U07413] and Hartmannella vermiformis [GenBank:AF426157] were used as outgroup (not shown). [file 2046-9063-9-16-S3.png]

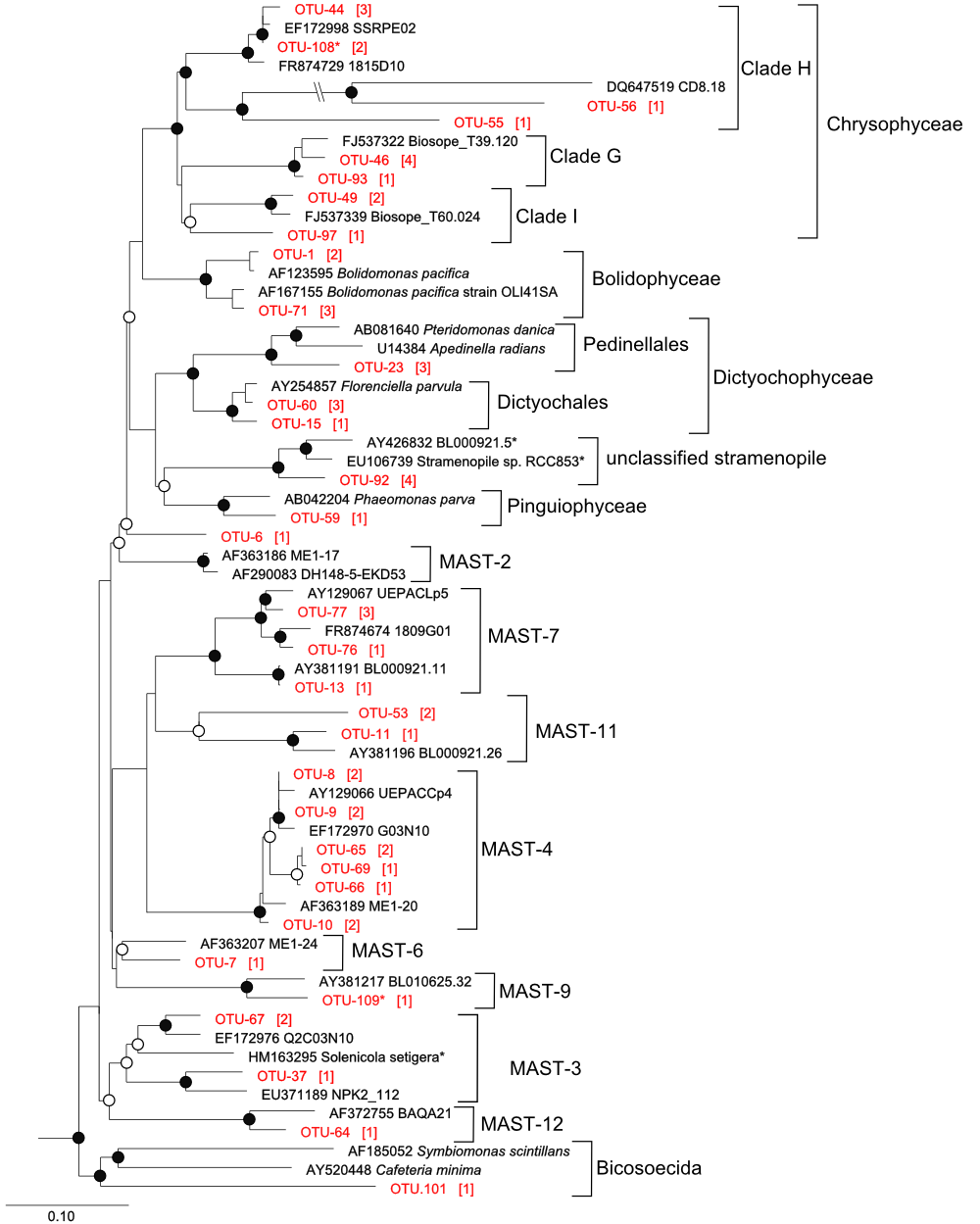

Supplement: Additional file 4: Figure S4 — Phylogenetic tree of representative stramenopile OTUs. Sequences from this study are shown in red color, with the number in brackets denoting the sequences counts per OTU. Bootstrap values for both ML and NJ methods are indicated at the branch nodes as open (>50%) and closed circles (>90%). An asterisk denotes partial 18S rDNA sequences. Sequences from Acanthamoeba castellanii [GenBank:U07413] and Hartmannella vermiformis [GenBank:AF426157] were used as outgroup (not shown). [file 2046-9063-9-16-S4.png]

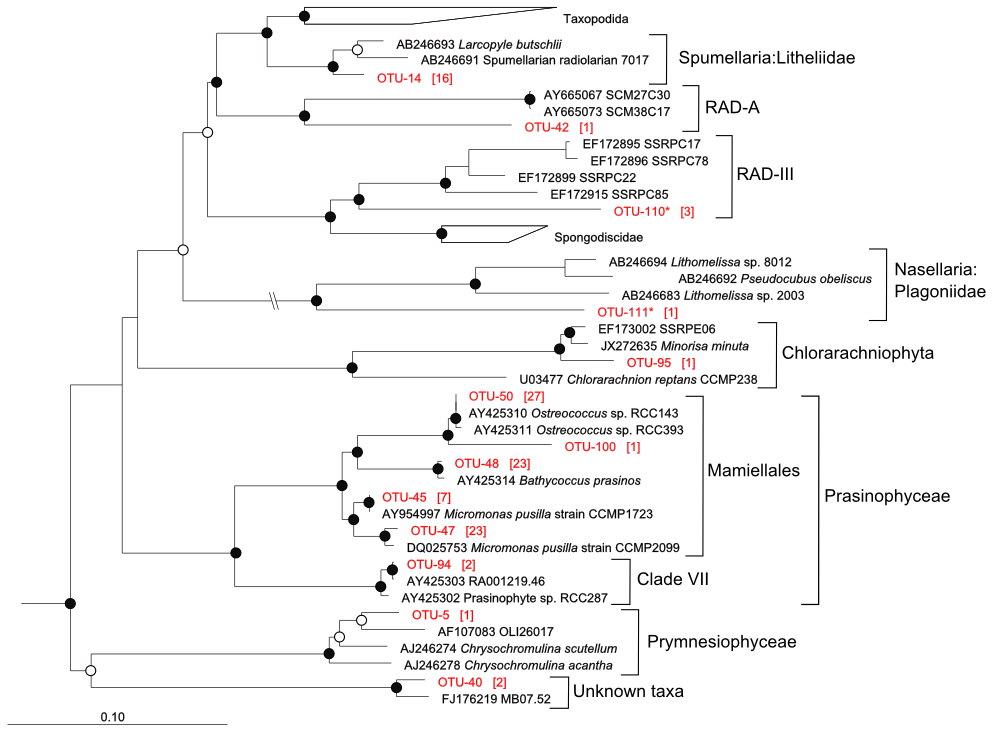

Supplement: Additional file 5: Figure S5 — Phylogenetic tree for representative eukaryotic OTUs not related to alveolates or stramenopiles. Sequences from this study are shown in red color, with the number in brackets denoting the sequences counts per OTU. Bootstrap values for both maximum likelihood and neighbour-joining methods are indicated at the branch nodes as open (>50%) and closed circles (>90%). An asterisk denotes partial 18S rDNA sequences. Sequences from Acanthamoeba castellanii [GenBank:U07413] and Hartmannella vermiformis [GenBank:AF426157] were used as an outgroup (not shown). [file 2046-9063-9-16-S5.png]
